# The utility of Aspirin in dukes C and high risk dukes B colorectal cancer - The ASCOLT study: study protocol for a randomized controlled trial

**DOI:** 10.1186/1745-6215-12-261

**Published:** 2011-12-14

**Authors:** Raghib Ali, Han-Chong Toh, Whay-Kuang Chia

**Affiliations:** 1 INDOX Cancer Research Network,, Richard Doll building, University of Oxford, OX37LF, UK; 2 Department of Medical Oncology, National Cancer Centre, 11 Hospital Drive, Singapore 169610

**Keywords:** ASCOLT, aspirin, platelets, colon, rectal, cancer, inflammation, adjuvant, Dukes B, Dukes C

## Abstract

**Background:**

High quality evidence indicates that aspirin is effective in reducing colorectal polyps; and numerous epidemiological studies point towards an ability to prevent colorectal cancer. However the role of Aspirin as an adjuvant agent in patients with established cancers remains to be defined. Recently a nested case-control study within the Nurses Health cohort suggested that the initiation of Aspirin *after *the diagnosis of colon cancer reduced overall colorectal cancer specific mortality. Although this data is supportive of Aspirin's biological activity in this disease and possible role in adjuvant therapy, it needs to be confirmed in a randomized prospective trial.

**Methods/Design:**

We hypothesize through this randomized, placebo-controlled adjuvant study, that Aspirin in patients with dukes C or high risk dukes B colorectal cancer (ASCOLT) can improve survival in this patient population over placebo control. The primary endpoint of this study is Disease Free Survival and the secondary Endpoint is 5 yr Overall Survival. This study will randomize eligible patients with Dukes C or high risk Dukes B colorectal cancer, after completion of surgery and standard adjuvant chemotherapy (+/- radiation therapy for rectal cancer patients) to 200 mg Aspirin or Placebo for 3 years. Stratification factors include study centre, rectal or colon cancer stage, and type of adjuvant chemotherapy (exposed/not exposed to oxaliplatin). After randomization, patient will be followed up with 3 monthly assessments whilst on study drug and for a total of 5 years. Patients with active peptic ulcer disease, bleeding diathesis or on treatment with aspirin or anti-platelet agents will be excluded from the study.

**Discussion:**

This study aims to evaluate Aspirin's role as an adjuvant treatment in colorectal cancer. If indeed found to be beneficial, because aspirin is cheap, accessible and easy to administer, it will positively impact the lives of many individuals in Asia and globally.

**Trials Registration:**

Clinicaltrials.gov: NCT00565708

## Background

Colorectal cancer is the third most common cancer worldwide with almost 1 million new cases diagnosed each year. It is now also the third leading cause of cancer mortality in men and women with more than half of diagnosed patients dying from the disease [1]. Over the past 3 decades, the age-standardized incidence rate for colorectal cancer has increased two to fourfold in Asian countries such as China, Japan, South Korea and Singapore [2]. Mortality rates in Asian countries have risen concomitantly and in Singapore colon cancer has recently surpassed lung cancer as the commonest cancer with incidence rates now amongst the highest in Asia [3].

With the rising rates of cancer, a fundamental shift of the cancer burden has also occurred between the developed and developing world. This problem, highlighted in the 2008 WHO World Cancer Report, warned of a disproportionate number of cancer deaths occurring in developing countries. Currently of the annual 12 million new cancer cases diagnosed and the 7.6 million cancer deaths worldwide; 5.6 million new cases and 4.7 million cancer deaths occurred in developing countries [4]. By the year 2020, it is predicted that changes in the demographics of the population in developing nations will lead to approximately 70% of all new cancers occurring in lower income countries [5]. Efforts therefore will have to be made to develop novel therapies that are not only effective but also accessible to the people who need them [6].

### Adjuvant chemotherapy

Adjuvant 5-FU based chemotherapy has been proven to improve relative overall survival in duke's C colon cancer by approximately 25-35%, in a series of landmark clinical trials [7-11] conducted over the past 3 decades - and has become standard therapy following surgery for colorectal cancer.

In 2004, the MOSAIC study was able to establish a new standard of care. For the first time, a new agent (oxaliplatin), was shown to improve 3 year disease free survival (DFS) over infusional 5FU alone [12]. After 7 years of follow up, oxaliplatin was associated with an absolute 2.5% survival gain for Dukes C colon cancer. Although Oxaliplatin-5FU combinations have become the new standard of care for Dukes C colon cancer; neurotoxicity and thrombocytopenia continue to remain significant challenges in the clinic.

Since the MOSAIC study was published in 2004, no other new agents have been shown to improve colon cancer outcomes. For example, although highly effective in the metastatic setting, Irinotecan chemotherapy has failed in three large randomised adjuvant studies - the EORTC PETACC-3, ACCORD and CALGB 89803 [13-15]. The failure of irinotecan is particularly disconcerting, since there are so few agents with a similar track record of efficacy in the metastatic setting. However, the consistent negative results in these high profile studies, means that further trials with Irinotecan are unlikely to be undertaken in the future [16]. With conventional chemotherapy combinations appearing to be reach their therapeutic index, strategic focus shifted to biological therapies including monoclonal antibodies to Vascular Endothelial Growth Factor (VEGF) and Epidermal Growth Factor Receptor (EGFR).

### Biological Therapies

The idea of using anti-VEGF monoclonal antibodies such as Bevacizumab, upfront in the adjuvant setting is one that carries particular scientific merit. The concept of the "angiogenic switch" means that tumours more than 1 mm would need to eventually grow their own blood supply in order to survive [17]. Consequently, bevacizumab should theoretically be even more effective, when used to treat tumours that are small, and particularly vulnerable to vascular disruption. Two large adjuvant bevacizumab studies have been undertaken to prove this concept - the NSABP C08 trial and the AVANT study. However, the results of the NSABP-C08 study published in 2011 was negative for its primary endpoint. More than 2000 patients were randomised to either 5FU/Oxaliplatin or the same chemotherapy with Bevacizumab and after a median follow up of 3 years, there was no significant difference in disease free survival between the 2 populations [18].

Cextuximab, a chimeric human-mouse monoclonal antibody to VEGF receptor is currently under development as an adjuvant agent for colon and rectal cancers. Although Cetuximab is highly effective in the metastatic setting, the preliminary results of the NCCTG Intergroup 0147 study evaluating its use in the adjuvant setting for kras mutated and wild-type tumors - indicates that it neither improves disease free survival nor overall survival [19].

### Cox 2 specific inhibitors

Since the discovery that cox-2 enzyme was consistently over-expressed in colon cancer; a large body of pre-clinical scientific evidence has emerged implicating cox and PGE2 in cancer initiation and propagation [20,21]. This has culminated in two large randomised trials evaluating Cox-2 specific inhibitors as specific adjuvant agents in colon cancer - VICTOR and the EORTC PETAAC 5. Both these trials were discontinued prematurely following concerns about the cardiovascular safety for these agents when in extended use. PETAAC 5 was a double blind randomised placebo controlled study, and evaluated celecoxib or placebo for 3 years and used DFS as a primary endpoint [22].

Launched in 2001, VICTOR randomised patients with stage II or III colon and rectal cancer that had completed standard adjuvant therapy to 2 years of rofecoxib, 5 years of rofecoxib or placebo, before it was prematurely suspended in 2005 when rofecoxib was withdrawn from the market [23]. In contrast to the EORTC study, VICTOR only randomized patients after completion of standard adjuvant therapy and it also included rectal cancers and stage II disease. In the analysis of 2300 patients who had been treated with study drug for a median of 7 months, there was no difference observed between the two treatment groups in terms of disease free survival and overall survival [24]. However due to the short median exposure to study medication and the failure to achieve accrual target, it is not possible to make any conclusion concerning efficacy.

### Aspirin

The first suggestion that NSAIDs/Aspirin may be beneficial in the adjuvant setting derives from a pre-planned review of patients in the CALGB 89803 study. This study randomised stage III colon cancer patients to 5FU chemotherapy with or without irinotecan, and in addition to the primary study, incorporated a pre-planned analysis to compare outcomes of patients who were on Aspirin or cox2 inhibitors. Of the 830 patients surveyed, 75 patients and 41 patients used Aspirin and Cox-2 inhibitors regularly. Amongst Aspirin and Cox2 users, the hazard ratio for disease recurrence was 0.45 (95% CI 0.21-0.97) and recurrence and/or death 0.48 (95%CI 0.24-0.99). No difference however was noted with paracetamol use [25].

More recently, a nested case control study within the Nurses Health Study) cohort suggested that the initiation of Aspirin *after *the diagnosis of colon cancer was able to reduce colorectal cancer specific mortality (HR 0.53, CI 0.33-0.86) on multivariate analysis [26]. Regular aspirin use after diagnosis was associated with an impressive lowering of colorectal cancer-specific mortality among participants in whom primary tumors overexpressed COX-2 (multivariate HR, 0.39; 95% CI, 0.20-0.76). This is in contrast to patients with tumours that had weak or absent cox2 expression where aspirin use was not associated with lower risk (multivariate HR, 1.22; 95% CI, 0.36-4.18). This suggests a biologically plausible mechanism for aspirin's activity.

Although the results from both these studies are extremely exciting and have the potential to open up new avenues in our understanding of cancer biology; they are observational studies and so may be due to bias and confounding. Therefore these findings need to be evaluaed in a prospective randomized placebo-controlled study which is the the aim of the ASCOLT study.

## Methods/Design

### Objectives

The primary endpoints for this study are Disease Free Survival for all eligible patients (colon, rectal, dukes C colon, high risk Dukes B colon, and Rectal cancer combined) and Disease Free Survival for colon cancer subjects (Dukes C and high risk Dukes B colon cancer). The secondary endpoint for this study is 5 year Overall Survival.

### Study population and design

This is a double blind, placebo-controlled, randomized study investigating aspirin in Duke B and C colorectal cancer. Patients who have complete resection of their primary tumour and who have completed standard adjuvant therapy (chemotherapy ± radiotherapy) within 90 days, and without bleeding diathesis or contraindication to aspirin will be eligible for the study. Patients on aspirin, anti-platelet therapy, anti-coagulation, ischemic heart disease, stroke, peripheral vascular disease or uncontrolled hypertension will be excluded from this study. Adjuvant chemotherapy is not specified but should consist of at least 3 months of a 5FU based chemotherapy. Rectal cancers may additionally have radiotherapy administered in either adjuvant or neoadjuvant fashion. Eligible subjects will be randomised to the study in a 1:1 ratio to either Aspirin 200 mg or matching placebo once daily for 3 years. Patients will be randomised in a competitive recruitment process from participating centres over 5 years. Randomisation will be done via direct web randomisation, and patients will be stratified by study centre, tumour type (Dukes C colon, high risk Dukes B colon cancer & rectal cancer sub-groups) and type of adjuvant chemotherapy received (exposed/not exposed to oxaliplatin). The steps for direct web randomization are as follows:

1. Authorized study centre personnel will randomise the patient via a password-protected internet web site http://randomise.cteru.com.sg.

2. The following information will be entered and the patient will be stratified by:

- Study centre

- Tumour type (Dukes C colon, high risk Dukes B colon cancer & rectal cancer sub-groups)

- Type of adjuvant chemotherapy received (exposed/not exposed to oxaliplatin)

3. The randomisation system will then determine the treatment arm and provide the subject number to be used for the patient.

4. The site monitor/CRA will be informed immediately in the event that the web randomisation is not successful.

**Figure 1:**
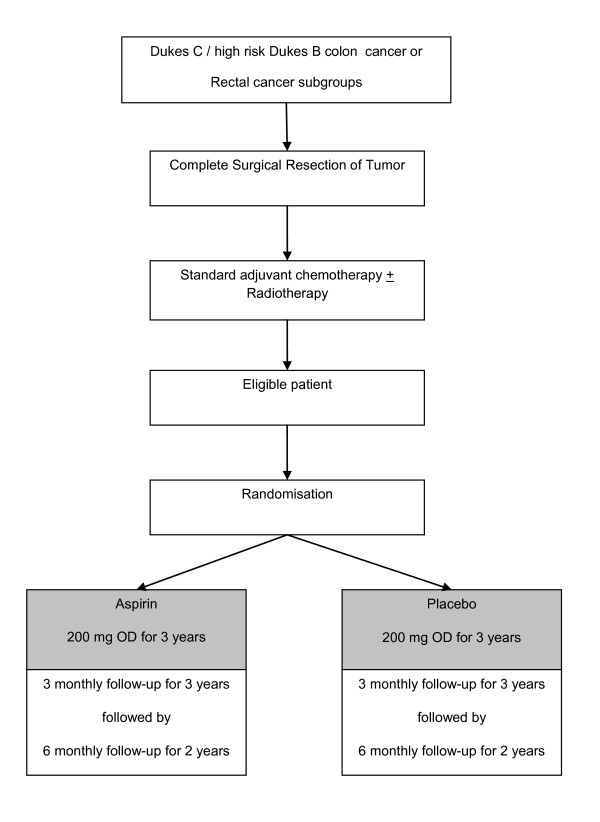
**CONSORT Diagram**. OD, once daily.

After randomisation, patients will have 3 monthly assessments for 3 years (month 3 to month 36) followed by 6 monthly assessments for additional 2 years (Figure 1).

### Endpoint assessment

Disease recurrence is defined as any one of the following - unequivocal radiological evidence of colorectal cancer recurrence, recurrence detected by digital rectal examination (DRE), positive histology or cytology (i.e. peritoneal or pleural cytology), colonoscopic evidence of local cancer recurrence at the previous operation site, detection of a new colon or rectal primary tumour. Disease free survival is defined as the time from randomisation to the time of disease recurrence or death from any cause and overall survival is defined as the time from randomisation to the time of death from any cause. Compliance is defined as taking the study drug for more than 70% of days during each scheduled follow-up visit throughout the treatment duration of 3 years.

### Sample size calculation

The total trial size will be 2660 patients, 1330 randomised to Aspirin group and 1330 randomised to Placebo group. In the sub-groups, there should be at least 2000 high risk Dukes B or Dukes C colon cancer patients, others are rectal cancer patients

It is assumed that 3-years disease free survival rate for Dukes B colon cancer, Dukes C colon cancer and rectal cancer are 65% after standard adjuvant chemotherapy; and the attrition rate is 5%. Therefore, the total trial size (2660) will be sufficient to detect a 6% absolute difference of disease free survival rate for all subjects between the two treatments, with a two-sided logrank test of 5% type I error and 90% power; further, our 3-year DFS rate for colon cancer is assumed to be 65%, which is similar to the entire group as a whole, the size of colon cancer (2000) will be sufficient to detect a 6% absolute difference of disease free survival rate for colon cancer between the two treatments, with a two-sided logrank test of 5% type I error and 80% power.

### Statistical Analysis

All statistical analyses will be carried out on an intention-to-treat basis. In the analysis of disease free survival, a patient is considered to have an event if he/she relapses after randomisation. The starting point for disease free survival is the date of randomisation and the terminating point is the date of first relapse or date of death, whichever occurs first. Patients in whom there has been no evidence of disease after treatment are censored at the date of last follow-up. Similarly, the overall survival time is computed from the date of randomisation to the date when the patient is last known to be alive.

Survival curves will be constructed using the Kaplan-Meier method and life table estimates of 3 and 5 year survival rates will be calculated. The efficacy of Aspirin will be estimated by the HR and its corresponding 95% CI and the Cox proportional hazards model will be used to adjust the HRs for the trial stratification factors (site, type of tumour and type of adjuvant chemotherapy). Stratified analysis and other non-proportional hazard models (which allow for the effects of covariates to vary over time) would be considered when the proportional hazards assumption is not valid. This assumption will be checked by using graphical methods and statistical tests during the modelling. Results from non-proportional models such as stratified Cox model or Cox model with time-dependent covariates will then be reported as the main analysis (depending on the appropriateness of each model to the actual data).

The secondary endpoint will be analysed in a similar manner to the primary endpoints. Tests for interaction for the colon cancer subgroups and other subgroups will be conducted. Analyses of primary and secondary endpoints will be repeated within the subgroups defined by ethnicity and tumour type. Analysis of primary and secondary endpoints will also be repeated for compliant and non-compliant subjects. In addition, cumulative incidence function will be estimated and the two treatment arms will be compared using Gray's method if competing risks are present.

### Interim analysis

An independent data and safety monitoring committee (DSMC) will be established to review the interim results of the study. Two interim analyses are scheduled. The first interim analysis should be done after 540 patients have been recruited (estimated to take between two to three years) or mid-point of the targeted recruitment period, and the second interim analysis should be done once 540 patients have been followed up for 3 years (approximately between five to six years). The aim of the first interim analysis is toxicity whereas the aim of the second interim analysis is disease free survival and overall survival as well as toxicity profile. The results of the second interim analysis will not be the sole criteria for deciding whether to terminate accrual or report the results early. Rather they will provide a guideline to aid in the decision, which will also take into account the characteristics of the patients, nature of toxicities, relevant external results. Most importantly, the sample size will be re-estimated. Apart from the reason of safety, which may caution otherwise, the minimum trial size will remain as 2660 as initially planned.

### Anticipated side effects of Aspirin

Side effects that occasionally occur are gastrointestinal disorders such as nausea, vomiting, diarrhoea, and slight gastrointestinal blood loss which in exceptional cases can lead to anaemia. Gastrointestinal ulcers may rarely develop, in some circumstances with haemorrhaging and perforation. Dizziness and ringing in the ear can occur as symptoms of overdosage, especially in children and elderly patients.

Rare cases of hypersensitivity reactions (e.g. difficulty in breathing, skin reaction) can occur as have isolated cases of liver and kidney function disturbances, and severe skin reactions have been reported. The absolute annual increase risk attributable to Aspirin for major bleeding, major gastrointestinal bleeding and intracranial haemorrhage has been estimated in a large meta-analysis to stand at 0.13%, 0.12% and 0.03% respectively [27].

### Blinding

The patient, the study team including the investigator(s) and the sponsor will be blinded. However, the study statistician who prepares the randomisation list and the designated unblinded personnel who prepare the study drug packaging and labelling will not be blinded. The study statistician will prepare the sealed emergency code-break envelopes and distribute to the study centres accordingly. The envelopes will contain the treatment assignment, with the corresponding subject number printed on it, and will be kept at site by the investigator or designated person. At interim analysis, only the study statistician will be unblinded.

### Treatment modification

The study drug should be started immediately but no later than 2 weeks after randomisation. The patient will be treated with the same study drug for 3 years. Treatment may be modified according to guidelines provided in the study protocol and according to clinical judgement and best medical practices. Patients who need to undergo elective surgery or other interventional procedures may stop the study drug 5 days prior to surgery and recommence the study drug upon recovery (when haemostasis is secured or when the patient is able to take orally). Patients who develop anaphylaxis, angioedema or gastrointestinal bleeding should stop the study drug immediately and should not undergo re-challenge to study drug. Patients who are unable to tolerate 200 mg of study drug may have the dose reduced to 100 mg.. The reason and date of dose reduction will be clearly documented. Proton pump inhibitors (PPIs) will be used in patients who have symptoms of epigastric discomfort. They should be given at adequate doses and continued for at least 3 months. PPIs are preferable to H2 antagonists. Antacids (i.e. magnesium trisilicate, magnesium carbonate) should not be given in place of PPIs; however, they may be used to supplement these agents. Patients who undergo gastroscopy should be screened for helicobacter pylori and treated accordingly.

### Treatment discontinuation

Study drug will be stopped immediately if there is a disease recurrence confirmed by CT, histology or cytology. In the event of an SAE related to study drug, the study drug will be stopped immediately but patients will continue to be followed up until 5 years after randomisation.

### Assessment and follow-up

Patients will begin screening after the last dose of standard therapy (chemotherapy ± radiotherapy). This will consist of written informed consent, medical history (asthma, diabetes, ischaemic heart disease, stroke, gastrointestinal ulcers or bleeding, alcohol history, smoking), family history of colorectal cancer, current medication, vital signs (body weight, height, BP), and blood investigations within 4 weeks of randomization (haematology, urea, creatinine, liver function tests, CEA). Full colonoscopy, CT abdomen ± pelvis, Chest X'Ray (or CT thorax) and ECG will be valid if performed within 12 months prior to screening.

Patients will be followed up at 3-monthly intervals whilst on study drug (i.e. month 3, 6, 9, 12, 15, 18, 21, 24, 27, 30, 33, and 36). Concomitant medication, records of adverse events (AE) and severe adverse events (SAE), CEA, haemoglobin, and surveillance colonoscopy (month 6 and 30) and surveillance CT scans (month 6, 18 and 30) will be recorded during visits. Following completion of study drug, patients will be assessed at 6-monthly intervals (month 42, 48, 54 and 60).

## Discussion

This study represents the first randomized aspirin trial, and a novel therapeutic approach, in the treatment of established colorectal cancer. The failure of 3 large randomized trials to show benefit for irinotecan in the adjuvant treatment of colorectal cancer and the recent disappointing failure of anti-VEGF and anti-EGFR monoclonal antibodies to improve survival makes the search for effective new agents all the more urgent.

Although substantial clinical, epidemiological [28-33] and pre-clinical data [34-44] points to a possible biological role for NSAIDs, and in particular Aspirin, in influencing the progression of colorectal cancer - to date no clinical trial of Aspirin in the adjuvant setting has been undertaken. Two large randomized studies exploring cox-2 inhibitors, the EORTC PETACC and the VICTOR trial had been undertaken, but were suspended prematurely, due to concerns of cardiovascular toxicity. More recently a new study by the CALGB exploring celecoxib in Stage 3 colon cancer has been initiated in US. Nevertheless, concerns still remain regarding cardiovascular toxicity in prolonged use. Aspirin by contrast is a non-selective cox inhibitor and is cardio-protective - and one of the most widely used drugs with a long history of use. If aspirin is indeed found to be beneficial, because it is cheap and easy to administer, it may have a larger impact for many patients in Asia and globally.

The optimal dose of Aspirin as an adjuvant agent for colorectal cancer is not known. There have been, to date, no randomised Aspirin trials exploring *secondary prevention *as an endpoint. Non-randomised studies evaluating Aspirin as a *primary prevention *agent have suggested that a dose of 325 mg a day for 5 years is effective [32]. In the analysis of the Nurses Health Study, regular use of standard Aspirin (325 mg) twice or more per week was shown to reduce the incidence of colorectal cancers. In contrast, analysis from randomised cardiovascular and stroke trials did not support additional benefit above 75 mg [45]. In the polyp prevention study by Baron et al, a lower dose (81 mg) of Aspirin appeared to be at least equally as effective as an intermediate dose (325 mg) in preventing recurrence of polyps [46]. In addition, dose escalation studies in normal human subjects using mucosal PGE2 as a biomarker have suggested that 81 mg Aspirin dose was sufficient to significantly suppress rectal mucosal PGE2 levels and did so to an equivalent extent as higher doses [47,48]. Our study using a 200 mg daily dose, falling into the middle of the range of doses in earlier studies - reflects the ongoing uncertainty concerning optimal dose. Whilst recognizing that standard dose Aspirin (i.e. 325 mg OD) may be the most logical dose to pursue in this clinical trial, we feel that a 200 mg intermediate dose would be more tolerable in our predominantly Asian trial population.

To our best knowledge, this trial represents the first attempt to evaluate aspirin as an adjuvant agent in Dukes B and C colorectal cancer. If shown to be beneficial, the results of this trial will have a major impact on the management of a globally important disease.

## Trial Status

The trial was started in March 2009. To date more than 130 patients have been randomised. The study has been expanded to 35 sites.

## List of abbreviations

5FU: 5-Fluorouracil; AE: Adverse event; ASCO: American Society of Clinical Oncology; BP: Blood Pressure; CALGB: The Cancer and Leukemia Group B; CEA: Carcinoembryonic antigen; CT: Computer Tomography; DFS: Disease free Survival; DRE: Digital Rectal Examination; DSMC: Data Safety Monitoring Committee; ECG: Electrocardiogram; ECOG: Eastern Cooperative Oncology Group; EGFR: Epidermal Growth Factor Receptor; EORTC: European Organisation for the Research and Treatment of Cancer; FA: Folinic Acid; FOLFOX: FOLFOX is a chemotherapy regimen for treatment of colorectal cancer, made up of the drugs, folinic acid (FOL), fluorouracil (F) and oxaliplatin (OX); HR: Hazard Ratio; NSAID: Non-steroidal anti-inflammatory drugs; OS: Overall Survival; PGE2 Prostaglandin E2; PPI: Proton pump inhibitor; RT: Radiation therapy; SAE: Serious Adverse Event; VEGF: Vascular Endothelial growth factor; WHO: World Health Organisation.

## Competing interests

The authors declare that they have no competing interests.

## Authors' contributions

WKC, THC and RA developed the original protocol and drafted the manuscript. All authors read and approved the final manuscript.

## ASCOLT Investigators

The trial and publication is on behalf of the ASCOLT Investigators: **The Sixth Affiliate Hospital**: JP Wang, YH Deng, X Jian; **Foshan Hospital**: W Wang;**Yonsei Cancer Centre**: JK Roh, AJ Bae, SJ Shin; **Penang Adventist Hospital**: TA Raj, E Nathan; **Hospital Kuala Lumpur**: R Khong, F Lau, A Deniel; **Pantai Hospital KL**: J Low; **Pantai Hospital AK**: D Tan; **University Malaya Medical Centre**: GF Ho, BMY Mastura, AC Roslani; **University Kebangsaan Malaysia Medical Centre**: M Azrif; **Queen Mary Hospital**: T Yau, WL Law; **Dr Sardjito Hospital**: J Kurnianda, I Purwanto, K Widayati; **Dharmais National Cancer Centre Hospital**: A Soemardi, S Syafei; L Mellinas, R Andalusia, S Noorwati; **Cipto Mangunkusumo General Hospital**: I Basir, M Abdullah, F Maengkom, I Rinaldi, G B Prajogi; **Johns Hopkins Singapore IMC**: G Lopes, G Ku, L Bharwani, A Chopra, A Chang; **National Cancer Centre Singapore**: CK Tham, S Ong, SP Choo, SK Lo, WH Koo, HY Lim, I Tan, KH Lim. **Tan Tock Seng Hospital**: R Sim; **Kidwai Memorial Institute of Oncology**: L Dasappa; **Nizam's Institute of Medical Sciences**: G Sadashivudu; **Tata Memorial Hospital**: Mohandas Mallath; **All India Institute of Medicine**: A Sharma; **Christian Medical College**: RT Chacko; **GKNM Valavadi Narayanasamy Cancer Centre**: B Sivanesan, A Rajkumar; **Amrita Institute of Medical Sciences**: TS Ganesan; **Regional Cancer Centre**: A Sajeed; **King Fahad Medical City**: A Ismail, **Taipei Medical University Hospital**: CI Hsieh, PL Wei, LJ Juo; **TMU Shuang Ho Hospital**: TY Chao, CM Chen, YY Hsieh, TC Chang; **Wan Fang Hospital**: GM Lai, YW Su, CM Chou; **Taipei Vetrans General Hospital**: CC Yen, JK Lin, JH Liu, TC Lin, HW Teng; **Chang Hua Hospital**: HC Chen, CS Chang, SY Huang, CC Wang, SY Lin, CY Chung, JT Lin, SZ Hsu; **Koo Foundation Sun Yat Sen Cancer Centre**: IP Huang, CH Chen, CC Chen, KC Huang; **National Cheng Kung University Hospital**: JC Lee, PC Lin, WC Su, BW Lin, SC Lin; **Siriraj Hospital**: T Kullathorn, C Akewanlop, P Dankulchai, Y Chansilpa, T Akaraviputh; **King Chulalongkorn Hospital**: S Chucheep, PA Jirawat, P Atittharnsakul, K Tantiplachiva; **Phillipines General Hospital**: DL Sacdalan, D Parreno;
